# Insights into the present and future of cartilage regeneration and joint repair

**DOI:** 10.1186/s13619-021-00104-5

**Published:** 2022-02-02

**Authors:** H. Evenbratt, L. Andreasson, V. Bicknell, M. Brittberg, R. Mobini, S. Simonsson

**Affiliations:** 1 Cline Scientific AB, SE-431 53 Mölndal, Sweden; 2 Institute of Biomedicine at Sahlgrenska Academy, Department of Clinical Chemistry and Transfusion Medicine, University of Gothenburg, SE-413 45 Gothenburg, Sweden; 3 Cartilage Research Unit, University of Gothenburg, Region Halland Orthopaedics, Kungsbacka Hospital, S-434 80 Kungsbacka, Sweden

**Keywords:** Articular Cartilage, Osteoarthritis, Joint treatments, Stem cell therapy, Gradients, Differentiation, Chondrocyte characterization

## Abstract

Knee osteoarthritis is the most common joint disease. It causes pain and suffering for affected patients and is the source of major economic costs for healthcare systems. Despite ongoing research, there is a lack of knowledge regarding disease mechanisms, biomarkers, and possible cures. Current treatments do not fulfill patients’ long-term needs, and it often requires invasive surgical procedures with subsequent long periods of rehabilitation. Researchers and companies worldwide are working to find a suitable cell source to engineer or regenerate a functional and healthy articular cartilage tissue to implant in the damaged area. Potential cell sources to accomplish this goal include embryonic stem cells, mesenchymal stem cells, or induced pluripotent stem cells. The differentiation of stem cells into different tissue types is complex, and a suitable concentration range of specific growth factors is vital. The cellular microenvironment during early embryonic development provides crucial information regarding concentrations of signaling molecules and morphogen gradients as these are essential inducers for tissue development. Thus, morphogen gradients implemented in developmental protocols aimed to engineer functional cartilage tissue can potentially generate cells comparable to those within native cartilage. In this review, we have summarized the problems with current treatments, potential cell sources for cell therapy, reviewed the progress of new treatments within the regenerative cartilage field, and highlighted the importance of cell quality, characterization assays, and chemically defined protocols.

## Background

Osteoarthritis (OA) is the most common form of chronic joint disease, affecting all joints in the body, resulting in progressive cartilage degeneration. Risk factors associated with OA include age, obesity, family history, or trauma that has caused damage to the cartilage (Haq et al., 2003). Physical inactivity has also been shown to lead to cartilage degradation as joints require mechanical load and motion to maintain healthy cartilage structure and function (Sophia Fox et al., 2009). As cartilage is an avascular tissue with sparse cell density, it has poor regenerative capacity. Due to this, OA results in pain, dysfunction, and substantial healthcare costs (Hudetz et al., 2017; Hiligsmann & Reginster, 2013). In addition to these direct effects, the disease leads to an indirect economic burden for societies due to decreased productivity and premature disability (Hiligsmann & Reginster, 2013). Since age is a substantial risk factor (Haq et al., 2003), and that the global life expectancy continues to increase, OA-related costs will also increase with time. Therefore, the potential cost savings provided by a cure, or other better alleviation methods, will also be substantial given the high prevalence of people suffering from the disease worldwide.

Recent reviews discuss cell-based treatments of OA and cartilage defects with a different focus. Both Agarwal et al. and Wiggers et al. dive deep into clinical studies of cellular therapies for improved knee function and decreased pain (Agarwal et al., 2021; Wiggers et al., 2021). In their respective meta-analyses, Agarwal et al. show that such treatment may be effective, while Wiggers et al. concluded that there is limited evidence for a qualitative effect. The future of stem cell therapy is dependent on high-quality cartilage to repair damage to a greater extent than is possible today. Kamaraj et al. reviewed studies that used induced pluripotent stem cells (iPSCs) to produce high-quality cartilage and tested the effect in vivo (Kamaraj et al., 2021). They concluded that iPSCs offer a valuable source of cartilage for effective cell-based therapy and that comparability of study findings is of utmost importance, in line with the focus areas of this review. This review will present an overview of current and possible future strategies for cell-based treatments for OA and cartilage defects. It will address current progress within the regenerative medicine field. It will also address the need for robust protocols for generating stem cell-derived chondroprogenitors or chondrocytes and valid characterizations used in stem cell therapies.

## Cartilage

Articular cartilage is a highly specialized and avascular tissue that is the most common type of cartilage covering the surface of articular joints (Schmutzer & Aszodi, 2017). It consists primarily of water (65–80% of wet weight), collagen fibers (10–20% of wet weight and 60% of dry weight, where type II collagens represent 90–95% of the collagen fibers), and proteoglycans (10–15% of wet weight). It also contains smaller amounts of other molecules such as glycoproteins, hyaluronan, and various elastic fibers, which form a dense extracellular matrix (ECM) network (Sophia Fox et al., 2009). The specialized cell type within adult cartilage, chondrocytes, is non-proliferating, and the cell density is relatively low. Only about 2% of cartilage consists of chondrocytes, where matrix proteins constitute the rest of the dry weight (Sophia Fox et al., 2009). The main function of chondrocytes is metabolic regulation, i.e., synthesis and degradation of ECM proteins, mainly collagen type II and aggrecan (Frazer et al., 1994). There are two alternative RNA splicing of collagen type II, one long-chained (collagen type IIA) characteristically expressed in pre-chondrocytes, and one short-chained (collagen type IIB) expressed in mature chondrocytes (Nah et al., 2001). Aggrecan is the most abundant proteoglycan within cartilage, and it is essential to maintain structure and function in this tissue. Due to its linkage to hyaluronan, aggrecan provides a hydrated gel structure necessary for biochemical and mechanical function. Aggrecan synthesis and degradation are regulated and, therefore, not constant throughout life. The degradation is directly linked to cartilage erosion and diseases such as OA (Song et al., 2007).

## Current treatments for OA

Despite much ongoing research regarding OA, there is a lack of knowledge regarding disease-related biomarkers, disease mechanisms, and drug targets (Zhang et al., 2016). There is no existing drug-based disease-modifying therapy on the market, although potential drugs are currently under investigation. There is also no specific treatment for halting cartilage degradation (Fosang et al., 2003). Current treatments are patient-specific and depend on the levels of pain a patient experience. Treatments are focused on lifestyle modifications such as diet and physical activity, pain and inflammation-reducing drugs, interarticular drug treatments, cell transplantations, and if needed, entire joint replacements (Zhang et al., 2016). However, pain-relieving and anti-inflammatory medications do not prevent the progression of the disease. Since surgery is an invasive procedure followed by long rehabilitation periods, it is normally only recommended for patients with a severe pain history.

## Surgical methods

Limb malalignment induces stress on articular cartilage, and when present in early OA, such malalignment causes further loss of articular cartilage. Unloading osteotomies can be used to realign the limb, reduce stress on degenerative cartilage, and to slow down disease progression. Osteotomies are used primarily for the knees, and can be used as a preservative tool for the joint (Mina et al., 2008). Joint distraction is a more recent technique where the bones are pulled apart to increase space, and the distracted area is fixed using pins combined with an external frame. It temporarily unloads the degenerative region, and the method has been used for OA conditions in the ankle and knee. Joint distraction appears to give patients short-term clinical and structural benefits with sustained effect up to 9 years (Goh et al., 2019).

Painful subchondral cysts in OA can be treated by subchondral plasty, filling the cysts with calcium phosphates and/or bone marrow concentrates (Szwedowski et al., 2020).

Joint replacements are a standard procedure with a limited lifespan that is used as late as possible in OA treatment. These procedures incur large costs for patients, hospitals, and healthcare systems. According to Chen et al., the money spent on joint replacements in the US alone has increased from ca 7 billion dollars in 1997 to over 22 billion dollars in 2004, with no sign of slowing down (Chen et al., 2012). With this in mind, we will focus on future alternative ways to treat OA.

### Autologous Chondrocyte Implantation (ACI)

Brittberg et al. developed an alternative method to treat local cartilage defects in the knee joints; autologous chondrocyte implantation (ACI). It involves harvesting the patient’s cells from a healthy and non-weight-bearing donor site, the isolation of chondrocytes, i.e., ECM removal, and cell expansion *ex vivo* to a sufficient number of cells. The cells are then implanted into the damaged area as a cell suspension, covered with a periosteal flap harvested from the patient’s tibia (Brittberg et al., 1994). It is, therefore, a two-step surgical procedure. The first trial was performed in humans in 1987 with good clinical outcomes and long-term follow-up (Peterson et al., 2010). Today, the method is widespread and used by surgeons worldwide (Ogura et al., 2017). This first-generation approach has evolved, first by the replacement of the periosteal flap with a collagen membrane (generation II) and then later to cells being grown on a cell carrier (generation III) such as matrix-assisted chondrocyte implantation (MACI) (Brittberg et al., 2018), or in a porous scaffold such as Hyalograft (Tognana et al., 2007). After culture, the cell-seeded scaffold is implanted into the defect (Gille et al., 2016). Scaffolds for tissue-engineered cartilage defects are commonly generated from biodegradable natural or synthetic biopolymers. Examples of scaffold materials for this purpose include cellulose, polycaprolactone, hyaluronan, collagens, as well as hydrogels such as agarose and alginate (Nguyen et al., 2011; El-Sherbiny & Yacoub, 2013). One strategy is to mix gels with rigid materials to create a more rigid scaffold. Liu et al. created a polycaprolactone/gelatin surrounded scaffold to enhance chondrogenesis of mouse iPSCs *in vitro* and *in vivo* with a promising outcome (Liu et al., 2014). Several studies have independently reported successful clinical outcomes of the cell-seeded implant approach using arthroscopy for implantation. (Gille et al., 2016; Basad et al., 2015). Although positive clinical outcomes are evident, the two-step surgical implantation process both involves the risk of limited access to autologous chondrocytes, as well as and their harvesting at a healthy donor site resulting in additional injury. Due to the cell expansion in monolayers, chondrocytes tend to dedifferentiate and change phenotype, which affects the synthesis of cartilage-specific matrix proteins essential for regeneration of the implanted chondrocytes (Watt, 1988).

One-stage 4th generation ACI techniques are emerging, and they are increasingly being implemented. Examples include mixing directly isolated chondrocytes with either directly isolated autologous bone marrow stem cells or allogeneic stem cells (Słynarski et al., 2020; de Windt et al., 2017). Particulated or fragmented autologous or allogeneic cartilage as a source for chondrocytes is also regarded as a 4th generation ACI. From crushed cartilage, the most active chondrocytes may migrate out into a surrounding supportive scaffold, gel, or similar (Cole et al., 2011; Grawe et al., 2017).

### Microfracture

An alternative surgical technique for treating local cartilage lesions uses bone marrow stimulations (BMS) such as microfracture. This arthroscopic technique creates small microfractures in the bone under the cartilage defect to trigger a regenerative response from mesenchymal stem cells (MSCs) in the bone marrow. The method is best suited for smaller defects created by trauma and not for OA (Lee et al., 2013). Additionally, younger patients (30–40 years old) have shown better outcomes than older patients (Knutsen et al., 2004). This procedure is relatively quick and cost-effective, as well as less invasive than ACI or joint replacements. However, the quality of the repaired MSC-derived cartilage exhibits variations between individuals.

Moreover, high-quality collagen type II-rich hyaline cartilage seems difficult to achieve, and a collagen type I-rich fibrous or hypertrophic cartilage is more likely to be generated (Saris et al., 2008). Despite this, the fibrocartilage might decrease symptoms in the affected joint and reduce pain for the patient. Local chondral and osteochondral lesions are mostly of traumatic origin, while osteoarthritis is an organ disease. A local cartilage lesion, if not treated, may increase in size and lead to OA. For local cartilage lesions, the choice of treatment is mainly based on the size of the lesion. A suggested cartilage lesion local treatment choice is presented here (Brittberg, 2021):

- BMS for small defects 0.5 cm^2^.
- Augmented BMS for small-medium sized defect 0.6–2 cm^2^.
- Augmentation is also an alternative for re-operations in such defects if a simple BMS has been done previously.
- Cell based treatments for large defects >2 cm^2^.
- Cell based treatments for re-operations >1 cm^2^.
- Osteochondral Allografts for extra-large defects.

## The drawbacks and possibilities of stem cell origins

The quality of the cells involved is one drawback of current surgical methods. Researchers are exploring other suitable cell sources that overcome the drawbacks of using autologous-derived chondrocytes to create a functional and healthy hyaline cartilage. Embryonic stem cells (ES-C), MSCs, and iPSC are potential cell sources for understanding OA disease mechanisms and use in a cell therapy-based treatment. ESCs are pluripotent and can divide infinitely (Takahashi & Yamanaka, 2006). However, problems such as the formation of teratomas and immune rejection have been reported. Such issues complicate the use of ESCs in regenerative medicine. Adult stem cell sources, such as MSCs that can be found in, e.g., bone marrow and adipose tissue, also have the potential to differentiate into several types of tissue (De Bari et al., 2001). The use of MSCs does not require immunosuppression, making them suitable for allogeneic cell banking as well as an off-the-shelf product (Huaman et al., 2019). They are also relatively easy to culture *in vitro*, as they do not tend to dedifferentiate like chondrocytes (Tallheden et al., 2004). However, MSCs have shown differing proliferation and differentiation capacity, depending on their tissue and molecular microenvironment origin (Maleki et al., 2014). Although MSCs have shown to be safe and efficient in pre-clinical studies, they have a tendency to form hypertrophic chondrocytes and bone instead of hyaline cartilage during chondrogenic differentiation, resulting in impaired biomechanical properties. A genetic discrepancy between articular and MSC-derived chondrocytes has also been detected. It was shown that MSC-derived chondrocytes resulted in a differing cartilage phenotype, and it was concluded that articular chondrocytes and MSCs differentiate along different pathways (Karlsson et al., 2007).

As MSCs are multipotent and can only differentiate into cells within the mesodermal lineage (Pittenger et al., 1999), an alternative cell origin is pluripotent stem cells. Like ESCs, iPSCs are pluripotent, have similar morphology and gene expression profiles, and can be divided infinitely (Takahashi & Yamanaka, 2006; Liu et al., 2010). iPSCs are a possible cell source with great potential within regenerative medicine and the treatment of cartilage defects and diseases such as OA. The use of iPSCs would overcome any present ethical issues surrounding ESCs, as they can be derived from a minimal number of easily accessible non-invasively harvested somatic cells. Mouse embryonic or adult fibroblasts were first induced to have pluripotency by Takahashi and Yamanaka in 2006 by using retroviruses. Since then, the required factors, Oct3/4, Sox2, Klf4, and c-Myc, have been used to induce pluripotency. These factors combined are known as the Yamanaka factors (Takahashi & Yamanaka, 2006). An adult cell can thus be reprogrammed back into the pluripotency developmental stage and be differentiated into any mature cell. This makes iPSCs useful in tissue engineering, regenerative medicine, drug screening, toxicity testing, and disease modeling.

One of the Yamanaka factors, C-Myc, is also a known oncogene, which is critical to consider when using iPSCs in clinical applications (Miller et al., 2012). Okita et al. showed how mouse fibroblasts were reprogrammed into iPSCs using the Yamanaka factors. They also discovered tumor generation in chimeric mice after cell transplantation due to the reactivation of the c-Myc gene (Takahashi et al., 2007; Nakagawa et al., 2010). Moreover, due to the pluripotency, there is a possibility that transplanted iPSCs form teratomas *in vivo*. Therefore, it is essential that no undifferentiated stem cells remain in the transplanted area (Liao et al., 2018). As mentioned, Yamanaka factors were first used to induce pluripotency via retroviruses. By using retroviral reprogramming, the virus’ RNA is converted to DNA and integrates with the donor cells’ cellular genome, which induces genomic change that can lead to unwanted gene transcription and increase the risk for tumor formation. Therefore, silencing the expression of Oct3/4, Sox2, Klf4, and c-Myc after reprogramming is essential to avoid harmful gene expressions. The use of retroviruses to induce pluripotency and the integrations with the cell genome makes this method unsuitable for human clinical applications (Takahashi & Yamanaka, 2006; Okita et al., 2007). To improve the reprogramming method, Okita et al. developed a viral-free method circumventing some of the above-mentioned side effects (Okita et al., 2008). A similar footprint-free method has been used to obtain large quantities of fully differentiated astrocytes from iPSCs (Mormone et al., 2014). Concurrently, Boreström, Simonsson et al. have shown that it is possible to eliminate the risk of genomic integrations or aberrations using a genetic footprint-free mRNA delivery system to induce iPSCs chondrogenic redifferentiation (Boreström et al., 2014). This discovery provides a significant step in the procedure to find a suitable cell source for regenerative medicine to treat, e.g., cartilage defects and OA.

The type and source of stem cells are only some aspects to consider when regenerating new and healthy hyaline cartilage. The cell microenvironment, biomolecular signaling, and other aspects of the differentiation process are equally significant issues that must be addressed. During early embryonic development, concentrations and signaling molecules in the cellular microenvironment are crucial, and morphogen gradients are essential inducers for all tissue development, including cartilage (Zecca et al., 1996; Dee et al., 2002; Jullien & Gurdon, 2005; Peret & Murphy, 2008). Differentiation into different tissue types can be complex, and the suitable concentration range of specific growth factors is critical (Dakhore et al., 2018). The morphogen gradients involved in the developmental process to engineer functional cartilage may be a potential tool for generating cartilage comparable to the function and strength of native cartilage. Using gradients as such a tool will be discussed further later in this review.

One controversial, due to the mentioned safety issues, question has been raised, especially applicable for cartilage regeneration; whether direct transplantation of iPSCs or committed cells at a certain differentiation stage would achieve better outcomes. While developmental immaturity of iPSC-derived cells can be a challenge for tissues like muscle and brain, Lee et al. demonstrate that it can be advantageous for cartilage (Lee et al., 2017a). This idea arises from the fact that particulated juvenile allograft cartilage (PJAC) transplantation has shown better long-term efficacy compared with, e.g., microfractures (Zhang et al., 2021; Adkisson et al., 2010). Nakayama et al. explore the possibility to treat cartilage lesions with iPSCs differentiated into juvenile chondrocytes, aiming to avoid the safety issues but letting the final differentiation to fully mature cells take place after transplantation (Nakayama et al., 2021).

## 3D Bioprinting as scaffolds for local repair

An upcoming strategy to improve the repair of local cartilage lesions is to use 3D bioprinting to generate a cartilage-like scaffold for the cells. Nguyen et al. concluded that a nanofibrillated cellulose composite bioink combined with alginate printed with human iPSCs and co-cultured with irradiated human chondrocytes was well suitable for bioprinting. This combination generated a cartilage-mimicking construct with cells expressing collagen II (Nguyen et al., 2017). One important goal that has yet to be reached with various scaffolds is to replicate the structural and biomechanical properties of native cartilage. 3D bioprinted MSC-containing hydrogels were used as constructs in an *in vivo* study in mice showing high structural integrity and good mechanical properties (Möller et al., 2017). Trials are also conducted *ex vivo,* where chondrocytes are 3D bioprinted *in situ* with promising results (Gatenholm et al., 2020). 3D-bioprinting is a hot topic and is discussed further in other recent reviews (Askari et al., 2021; Wu et al., 2021).

## Chondrocyte characterization and validation

To use stem cell-derived chondrocytes for cartilage regeneration *in vivo*, the characteristics of chondrocytes must be well-established. Different kinds of experimental setups such as immunoassays, histological assays, microarrays, quantitative polymerase chain reaction (qPCR), and fluorescent-activated cell sorting (FACS) are commonly used in combination with well-known chondrocyte markers such as collagen type II, SOX9, and aggrecan (Tallheden et al., 2004; Lach et al., 2019; Suchorska et al., 2017a). We reviewed articles featuring where ESCs, MSCs, or iPSCs differentiated into the chondrogenic lineage, as well as native chondrocytes, to understand how different research groups characterize chondrocytes and chondroprogenitors. Table 1 shows the four most commonly used experimental methods in the reviewed articles. Other methods used to a minor extent in the publications have been excluded from Table 1. Based on the reviewed articles, the most commonly used methods to characterize chondrocytes are qPCR, immunostaining, and histological staining that were often used in combination. FACS was used less than the other three assays in the studied articles to obtain supporting data or detect a study-specific marker.

**Table 1:** Characterization of chondrocytes or chondroprogenitors

Cell origin	qPCR	Immunostaining	Histology	FACS	Reference
ESCs	COL2, AGGRECAN, SOX9, SOX6, SOX5, COL9	COL2, SOX9	Safranin O, Hematoxylin and Eosin	SOX9	(Cheng et al., 2014)
ESCs	COL2, AGGRECAN, SOX9, SOX6, SOX5, CD44	COL2, SOX9		SOX9, CD44	(Oldershaw et al., 2010)
ESCs	COL2, AGGRECAN, SOX5	SOX9	Safranin O, Hematoxylin and Eosin	SOX9	(Wang et al., 2019)
MSCs	COL2, AGGRECAN, SOX9	COL2, chondroitin sulfate	Hematoxylin and Eosin, Alcian Blue		(Weissenberger et al., 2020)
MSCs	COL2, AGGRECAN, SOX9	COL2	Hematoxylin and Eosin, Alcian Blue, Safranin O	CD44	(Meng et al., 2016)
MSCs	COL2, AGGRECAN	COL2, AGGRECAN	Hematoxylin, Alcian Blue		(Lu et al., 2017)
iPSCs+ESCs	COL2, AGGRECAN, SOX9	COL2, SOX9, chondroitin sulfate	Safranin O, Alcian Blue van Gieson, Toluidine blue, Hematoxylin, and Eosin		(Lach et al., 2019)
iPSCs	COL2, SOX9, SOX6, SOX5	COL2, AGGRECAN, SOX9, SOX6, COL9, COMP		CD44, CD151	(Suchorska et al., 2017a)
iPSCs	COL2, AGGRECAN, SOX9, SOX6, COL9, COMP	COL2, AGGRECAN, SOX9, SOX6, COL9, COMP			(Suchorska et al., 2017b)
iPSCs	COL2, AGGRECAN	COL2, AGGRECAN	Safranin O		(Diederichs et al., 2019)
iPSCs	COL2, AGGRECAN, SOX9, COL9, COL11	COL2	Alcian Blue, Hematoxylin and Eosin		(Nejadnik et al., 2015)
iPSCs	COL2, AGGRECAN, SOX9	COL2	Safranin O, Alcian Blue van Gieson, Hematoxylin and Eosin		(Nguyen et al., 2017)
iPSCs	COL2, AGGRECAN, SOX9, SOX6, SOX5, LUBRICIN	COL2	Toluidine blue		(Rim et al., 2018)
iPSCs	COL2, AGGRECAN, COMP	COL2	Toluidine blue, Hematoxylin, and Eosin		(Wei et al., 2012)
iPSCs	COL2, AGGRECAN, SOX9	COL2	Safranin O, Hematoxylin	CD105, CD145, CD166, CD271	(Adkar et al., 2019)
iPSCs	COL2, AGGRECAN, SOX9	COL2, AGGRECAN	Alcian Blue, Toluidine blue		(Koyama et al., 2013)
Chondrocytes	COL2, AGGRECAN, SOX9		Alcian Blue van Gieson		(Enochson et al., 2014)
Chondrocytes		COL2	Safranin O, Alcian Blue van Gieson		(Tallheden et al., 2004)
Chondrocytes	COL2, AGGRECAN	COL2, AGGRECAN			(Naranda et al., 2017)
Chondrocytes	COL2, AGGRECAN, COMP		Toluidine blue, Hematoxylin, and Eosin		(Wei et al., 2012)

*Published articles were reviewed to overview how different research groups characterize their chondrocytes or chondroprogenitors originating from differentiated ES, iPSCs, MSCs, or chondrocytes. This table shows targeted genes and proteins in qPCR and immunoassays, histological stainings used, and which antigens were targeted with FACS.*

Many of the publications describe new or improved protocols for chondrogenic differentiation of stem cells. Some compare the level of gene expressions with adult chondrocytes (Lach et al., 2019; Suchorska et al., 2017a; Weissenberger et al., 2020; Suchorska et al., 2017b; Diederichs et al., 2019; Adkar et al., 2019; Koyama et al., 2013) . Others choose to compare the increase and decrease of markers within the study samples (Cheng et al., 2014; Oldershaw et al., 2010; Wang et al., 2019; Nejadnik et al., 2015). A high presence of the chondrogenic markers SOX9, COL2, and aggrecan is associated with high-quality articular cartilage regeneration. While the fibro- and hypertrophic cartilage markers, COL1A1, and COL10A1, respectively, should be low (Kamaraj et al., 2021). Also, SOX5, SOX6, COL9, and COL11 are well-known chondrogenic markers. Proteoglycans (Safranin O-staining), Glycosaminoglycans (Alcian Blue-staining), and immunohistochemistry staining for Collagen II are supportive in describing functional cartilage tissue. Other markers mentioned give additional supportive data, e.g., CD44 indicates normal chondrocyte function via connection to hyaluronic acid (Ishida et al., 1997), Hematoxylin and Eosin to visualize tissue cell structures, chondroitin sulfate is a chemical building block of cartilage, and lubricin and COMP indicates a functioning cartilage matrix (Flowers et al., 2017). During the differentiation process, the decrease in expression of pluripotency markers such as OCT4, Nanog, and SOX2 must be measured to ensure the absence of teratoma (Kamaraj et al., 2021).

Tissue engineering projects creating structures that should support the differentiation process can be evaluated using the same markers (Nguyen et al., 2017; Meng et al., 2016; Lu et al., 2017). The markers can also be used when comparing different cell origins after reprogramming them into iPSCs, and then differentiation towards chondrocytes (Rim et al., 2018; Wei et al., 2012). Additional uses are assessing the chondrogenic potential of cells isolated from patients, e.g., for ACI treatment (Tallheden et al., 2004; Naranda et al., 2017), and when studying the signaling pathways of chondrocytes (Enochson et al., 2014).

The characterization and validation are of significant importance to ensure cell specificity and quality. Obtaining high-quality cartilage repairing cells may be possible with an optimized protocol with defined cartilage-specific markers that can provide tight control over the resulting cell populations. There are advantages and drawbacks to consider depending on the choice of cell source (ESCs, MSCs, iPSCs, and chondrocytes), but all have a high potential for cartilage regeneration. We have reviewed different cell-based products for cartilage regeneration to summarize their current market status and ongoing clinical trials with current methods and problems in mind.

## Commercialization of new therapies

Worldwide, companies are focused on developing cell-based products that repair or regenerate cartilage to amend defects caused by, e.g., OA or trauma. Different strategies have been applied to accomplish this. The well-known ACI method has evolved to include a supporting matrix or scaffold product, aka matrix-associated autologous chondrocyte implantation (MACI). Recently, products that involved the administration of autologous or allogeneic stems cells through intra-articular injection have emerged, either with or without a supporting matrix. Another strategy is to surgically implant 3D biocompatible cell-seeded scaffolds, as described earlier in the review. However, cell-based therapies have been subject to strict regulation by authorities (Reisman & Adams, 2014) as well as logistical and production challenges. In Table 2, cell-based products that are currently approved or within clinical development for the treatment of cartilage damage are summarized.

**Table 2:** Products currently approved or undergoing clinical trials of cell-based products for cartilage repair

Product	Allogeneic /autologous	Market Status	Indication	Cell source	Delivery method	Clinical trial number	Ref.
**Chondrocytes, allogeneic**							
Invossa / TissueGene-C	Allogeneic	Phase III underway in the US, withdrawn in KR	Knee Osteoarthritis	Juvenile chondrocytes and transduced cells expressing TGF-B1	Intraarticular Injection	US Phase III NCT03203330	(Clinicaltrials.gov, 2017; Evans,^ 2019^)
**Chondrocytes, autologous**							
Chondron	Autologous	Approved in KR in 2001	Focal knee cartilage defect and arthritis	chondrocytes from patient joint	Cell suspension to be implanted with fibrin glue during arthrotomy	KR Follow up NCT01056900	(Ministry of Food and Drug Safety, 2019; Clinicaltrials.gov, 2010a)
JACC	Autologous	Approved in JP 2012	traumatic cartilage defect, Osteochondritis dissecans	chondrocytes from patient joint	Cells embedded in atelocollagen gel implanted with periosteal flap	JP study J-TEC002	(Ministry of Health, Labour and Welfare, 2012)
MACI	Autologous	Approved in US 2016, EU in 2013, withdrawn 2018	Articular Cartilage Defect	chondrocytes from patient joint	Cell-seeded collagen membrane secured with fibrin glue during mini-arthrotomy	US Phase III NCT00719576	(Food and Drug Administration, 2021; European Medicines Agency, 2018; Clinicaltrials.gov, 2008)
Ortho-ACI	Autologous	Approved in AU in 2017	Articular Cartilage Defects, knee, patella, ankles	Chondrocytes from patient joint	Cells and collagen scaffold implanted arthroscopy	None found	(Department of Health Therapeutic Good Administration, 2017)
Spherox	Autologous	Approved in EU 2017	Articular Cartilage Lesion of the Femoral Condyle	chondrocytes from patient joint	Spheroids of autologous matrix-associated chondrocyte implanted during arthroscopy or mini-arthrotomy	EU Phase III NCT01222559	(European Medicin Agency, 2021; Clinicaltrials.gov, 2010b)
Novocart 3D	Autologous	Approved in DE and CH in 2014, phase III in US and EU	Traumatic Articular Cartilage Defects in the Knee	chondrocytes from patient joint	Cells within bilayer collagen sponge implanted during arthrotomy	US Phase III NCT01957722 EU Phase III NCT01656902	(Paul Ehrlich Institut German Federal Ministry of Health, 2014; Swissmedic Swiss Agency for Therapeutic Products, 2014; Clinicaltrials.gov, 2013a)
CartiLife	Autologous	Approved in KR, Phase II underway in the US	Articular Cartilage Defect and Degeneration	Costal autologous chondrocytes	Pellet-cultured beads fixed with fibrin glue during arthrotomy	KR Phase II NCT03545269 Phase II US NCT04744402	(Ministry of Food and Drug Safety, 2019; Clinicaltrials.gov, 2021; Clinicaltrials.gov, 2018a)
**Stem Cells, allogeneic**							
Cartistem	Allogeneic	Approved in KR in 2012, Phase I/II a completed in the US	Cartilage Injury, Osteoarthritis	umbilical cord derived MSCs and sodium hyaluronate	During arthroscopy into holes drilled into defects	KR Phase III NCT01041001 US Phase I/II NCT01733186	(Clinicaltrials.gov, 2012; Clinicaltrials.gov, 2009)
CYP-004	Allogeneic	Phase III trial underway in AU	Knee OA	iPSC derived MSCs	Intraarticular Injection	AU Phase III ACTRN12620000870954	(Australian New Zealand Clinical Trial Registry, 2020)
Chondrogen	Allogeneic	Phase II underway in MY	Knee OA	Umbilical cord derived MSCs and HA	Intraarticular Injection	MY Phase II NCT04520945	(Clinicaltrials.gov, 2020a)
AlloJoin	Allogeneic	Phase II underway in CN	Knee OA	Adipose-derived mesenchymal progenitor cells	Intraarticular Injection	CN Phase II NCT04208646	(Clinicaltrials.gov, 2019a)
CELLISTEM-OA	Allogeneic	Phase I/II underway in CL	Knee OA	Umbilical-cord derived MSCs	Intraarticular Injection	CL Phase I/II NCT03810521	(Clinicaltrials.gov, 2019b)
Chondrochymal	Allogeneic	Phase I/II underway in TW	Knee OA	Bone marrow derived MSCs	Intraarticular Injection	TW Phase I/II NCT03589287	(Clinicaltrials.gov, 2018b)
Elixcyte	Allogeneic	Phase I/II underway in TW	Knee OA	Adipose-derived MSCs	Intraarticular Injection	TW Phase I/II NCT02784964	(Clinicaltrials.gov, 2016)
MAG200	Allogeneic	Phase I completed in AU	Bilateral Primary OA of Knee	Adipose-derived MSCs	Intraarticular Injection	AU Phase I ACTRN12617001095358	(Registration number ACTRN12617001095358, 2018)
Progenza	Allogeneic	Phase I completed in AU	Knee OA	Adipose-derived MSCs plus MSC secretions	Intraarticular Injection	AU Phase I ACTRN12615000439549	(Australian New Zealand Clinical Trial Registry, 2018)
SMUP-IA-01	Allogeneic	Phase I in KR completed	Knee OA	umbilical cord derived MSCs	Intraarticular Injection	KR Phase I NCT04037345	(Clinicaltrials.gov, 2019c)
**Stem Cells, autologous**							
JOINTSTEM	Autologous	Phase III completed KR, Phase II/III in US underway	Degenerative Arthritis Knee Osteoarthritis	Adipose-derived MSCs	Intraarticular Injection	KR Phase III NCT03990805 US PhaseII/III NCT04368806	(Clinicaltrials.gov, 2019d; Clinicaltrials.gov, 2020b)
AdMSCs	Autologous	Phase II underway in the US	Osteoarthritis, Knee, Hip, Shoulder	Adipose-derived MSCs	Intraarticular Injection and intravenous infusion	US Phase II NCT04448106	(Clinicaltrials.gov, 2020c)
ReJoin	Autologous	Phase II completed in CN	Knee Osteoarthritis	adipose-derived mesenchymal progenitor cells	Intraarticular Injection	CN Phase II NCT01809769	(Clinicaltrials.gov, 2013b)
Stemchymal OA Knee	Autologous	Phase I/II underway in TW	Knee OA	Adipose-derived MSCs	Intraarticular Injection	TW Phase I/II NCT02544802	(Clinicaltrials.gov, 2015)
PSC-01	Autologous	Phase I underway in the US	Knee OA	Adipose-derived MSCs	Intraarticular Injection	US Phase I NCT04043819	(Clinicaltrials.gov, 2019e)

*Products currently approved or undergoing clinical trials of cell-based products for cartilage repair. Products that are regarded as “minimally manipulated” and not subject to marketing approval are excluded. Products no longer on the market or with terminated clinical development are excluded. Product name, autologous or allogeneic, the most recent status of approval or clinical trials, the listed indication, cell source and delivery method of cells, and clinical trial numbers are provided where available.*

The earliest approved cell-based products of those reviewed are autologous chondrocyte implantation products. Of the chondrocyte-based products currently approved or in development, the majority are matrix-associated ACI products (JACC, MACI, Ortho-ACI, Spherox, Novocart 3D, Cartlife) where arthroscopically harvested chondrocytes are seeded within a matrix or scaffold material before implantation during a second procedure. These products have largely replaced previous product generations, which involved a liquid cell suspension and the use of a peristomal flap or a collagen membrane, such as Carticel and ChondroCelect (European Medicin Agency, 2017), both having been withdrawn from the market. Recent advances in this area have led to MACI products where cells are cultured to become more cartilage-like and include extracellular components. One example of this is Spherox, which was approved in the EU in 2017 (European Medicin Agency, 2021) following a Phase III clinical trial (NCT01222559) (Clinicaltrials.gov, 2010b). In this product, patient chondrocytes are condensed into spheroids, that is, spherical aggregates of ex vivo expanded chondrocytes with self-synthesized cartilage-specific extracellular matrix (Eschen et al., 2020). Also utilizing the ECM is Cartilife, which is approved in South Korea (Ministry of Food and Drug Safety, 2019) and is currently undergoing a Phase II clinical trial in the US (NCT04744402) (Clinicaltrials.gov, 2021). Cartilife uses costal-derived autologous chondrocytes, which are harvested, expanded, and then undergo a 3-dimensional pellet culture where cells form small beads with immature hyaline cartilage-like ECM (Lee et al., 2017b).

Of the reviewed chondrocyte-based products, there was only one that utilized an allogeneic cell source. Invossa is an intra-articular injection comprised of a combination of juvenile chondrocytes and cells transduced to express TGF- ß used in knee osteoarthritis. Recently, animal model studies into the potentially disease-modifying mechanisms behind the clinical results showed the treatment in rats caused an increase in anti-inflammatory cytokine IL-10 (Lee et al., 2020). The researchers suggest that the treatment improved OA through the structural improvement and analgesic effects of an anti-inflammatory microenvironment promoted by M2 macrophages, which are known to exhibit immunosuppressive properties within the knee joint (Lee et al., 2020). The product was approved in South Korea in 2017 but withdrawn in 2019. A Phase III Study is underway in the US (NCT03203330) (Clinicaltrials.gov, 2017).

### Stem cell-based products

An increasing number of products are emerging using stem cells such as MSCs and other progenitor cell types. In contrast to ACI, which more often focuses on focal defects, all the reviewed products are indicated for OA. For autologous stem cell products, common cell sources for MSCs are adipose tissue, bone marrow, and peripheral blood. The autologous adipose-derived MSC product JOINTSTEM recently completed a phase III trial (NCT03990805) (Clinicaltrials.gov, 2019d) in South Korea and is conducting a Phase II/III trial in the US (NCT04368806) (Clinicaltrials.gov, 2020b). Additionally, four companies currently are conducting Phase I or Phase II trials (Clinicaltrials.gov, 2020c; Clinicaltrials.gov, 2013b; Clinicaltrials.gov, 2015; Clinicaltrials.gov, 2019e)(NCT04448106, NCT01809769, NCT04043819, NCT02544802). A recent systematic review of randomized controlled trials (RCTs) for autologous stem cell therapy in knee osteoarthritis reviewed 14 RCTs and found a positive effect on patient-reported outcomes. However, they also reported a high risk of bias and low certainty of evidence (Wiggers et al., 2021).

Compared to the autologous stem cell products reviewed, a larger number of products in commercial clinical development were allogeneic. Allogeneic cell sources in Table 2 include adipose tissue, bone marrow, umbilical cord blood, and induced pluripotent stem cells. Allogeneic therapies have the advantage of being “off-the-shelf” as opposed to needing to source, transport, and process cells from a patient’s bone marrow or adipose tissue in the case of autologous therapy. As mentioned, it is generally accepted that MSCs can be used for allogeneic transplantations without the need for immunosuppression since the MSCs do not display immunogenic properties, which is a key advantage of using MSCs (Huaman et al., 2019).

The first allogeneic MSC product for cartilage injury, Cartistem, was launched in South Korea in 2012 (Ministry of Food and Drug safety, 2016), has conducted a Phase I/II trial in the US (NCT01733186) (Clinicaltrials.gov, 2012). The product combines allogeneic umbilical cord blood-derived MSCs and a hyaluronic acid hydrogel (Park et al., 2017). Unlike all the other reviewed stem cell products, which are intra-articular injections, Cartistem is administered through arthrotomy or arthroscopy with drilling (Park et al., 2017). Medipost, Cartistem’s developer, is currently developing a new generation product, an injectable MSC product, SMUP-IA-01, which has completed Phase I clinical trial in Korea (NCT04037345) (Clinicaltrials.gov, 2019c).

Cynata is currently conducting a Phase III study in Australia for CYP-004, an iPSC-derived MSC product (ACTRN12620000870954) (Australian New Zealand Clinical Trial Registry, 2020). Uniquely, CYP-004 is manufactured from iPSC cells through the intermediate step mesenchymoangioblasts (MCAs). iPSCs, as a cell source for cartilage regeneration, have some biosafety issues regarding the use *in vivo* discussed above. Eight other allogeneic stem cell products have completed or are undergoing Phase I or II studies, see Table 2.

## The importance of gradients in tissue-mimicking for stem cell therapy development

For decades, researchers have known about the importance of gradients in developmental biology (Zecca et al., 1996; Dee et al., 2002; Jullien & Gurdon, 2005; Peret & Murphy, 2008). Gradients are present in a wide range of biological processes *in vivo*, including development, inflammation, wound healing, and cancer metastasis. These processes can be studied *in vitro* using quantifiable and controllable gradients to mimic those present *in vivo*. In stem cell differentiation and development, the gradients are essential inducers of tissue structure generation and functionality (Zecca et al., 1996; Dee et al., 2002; Jullien & Gurdon, 2005; Peret & Murphy, 2008). The local gradients, consisting of biomolecules such as morphogens or growth factors, or physical characteristics such as stiffness gradients, are involved in cell regulation and the inducement of developmental processes (Zecca et al., 1996; Dakhore et al., 2018; Gurdon et al., 1994; Gurdon et al., 1998; Joaquin et al., 2016). Only a few articles have managed to visualize morphogen gradients *in vivo* or *in vitro* (Teleman & Cohen, 2000; Lagunas et al., 2013). However, as technology develops, different gradient setups have been increasingly employed to study stem cells. As gradient-regulated processes are present in various signaling systems throughout the cell surroundings, there are different approaches to how they are used depending on the aim of the study. It is also important to consider the scale and the level of precision available, from a macro scale down to influencing cells on a nano- or molecular level. The most studied gradual cell environment factors are stiffness, chemical/cell attachment, and biomolecular (e.g., morphogens, growth factors). Such studies aim to study migration, differentiation, cell proliferation, and growth optimization. The choice of approach varies and can overlap. Examples of types of gradients are hydrogels, microfluidics, nano-gradients, and plasma-treated polymer surfaces. Table 3 summarizes the literature on these approaches. There are drawbacks and benefits with all strategies, and in some cases combining techniques may be a successful alternative, depending on the aim of the study.

**Table 3:** Summary of gradient technologies and their use in cell applications

Technology	Hydrogel	Microfluidics	Plasma polymer/ polymer surface	Nano-gradient
Type of gradient				
**Stiffness**	Migration (Kim et al., 2015), Differentiation (Oh et al., 2016), Other cell behavior (Hadden et al., 2017; Idaszek et al., 2019)	NA	NA	No studies found
**Chemical/Attachment**	Other cell behavior (Idaszek et al., 2019)	NA	Differentiation (Liu et al., 2015; Wang et al., 2015)	No studies found
**Biomolecular**	Culturing/Growth (Mahadik et al., 2014), Migration (Addington et al., 2015), Differentiation (O’Grady et al., 2019; Smith Callahan et al., 2013), Other cell behavior (Idaszek et al., 2019)	Culturing/Growth (Mahadik et al., 2014), Migration (Won et al., 2014), Differentiation (O’Grady et al., 2019; Chung et al., 2005)	Culturing/Growth (Faia-Torres et al., 2015; Miller et al., 2011), Other cell behavior (Harding et al., 2012)	Differentiation (Andreasson et al., 2020a; Andreasson et al., 2020b)

*The reviewed literature is listed in the table to summarize the technology, type of gradient used, and what it was aimed to study. Gradient technology is indicated horizontally and gradient type vertically. The studied cellular responses are grouped into migration, differentiation, culturing/growth, and other cell behavior that vary significantly and are specific for each study.*

Regarding stem cell differentiation towards chondrocytes, little research is published around biomolecular gradients and their influence on differentiation despite the evident importance during tissue development (Jullien & Gurdon, 2005; Gurdon & Bourillot, 2001). The primary focus has been stiffness gradients based on mimicking the complex zonal microstructure of cartilage tissue. According to Idazec et al., current clinical treatments fail to regenerate new tissue that recapitulates this zonal structure resulting in the regenerated tissue lacking long-term stability (Idaszek et al., 2019). The study used a microfluidic printing device to shape gradients of chemical, mechanical, and biological factors into a layered cartilage-like structure in which MSCs and chondrocytes were co-cultured (Idaszek et al., 2019). This layered structure approach has been investigated and created in multiple ways using microfluidics, hydrogels, electrospun fibrous meshes, and cell sheets (Nguyen et al., 2011; Jin et al., 2019; Shi et al., 2013). Hydrogel stiffness gradients have also been used for investigating favorable stiffness ranges for induction of differentiation into specific cell types (Oh et al., 2016). All these techniques have their respective benefits and drawbacks depending on their use. However, they all aim to demonstrate how mechanical cues and loads control stem cell differentiation and tissue regeneration. Such studies are of great importance as it has been found that externally applied mechanical forces can stimulate stem cells to promote tissue regeneration (Enochson et al., 2014).

**Fig. 1:**
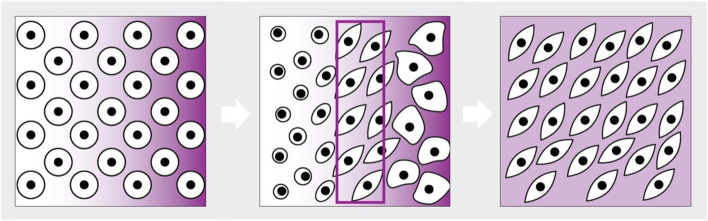
The figure is a schematic image of the use of gradient nanotechnology in cell differentiation. The cells are seeded on a gradient surface (left), and the differentiation process reveals an optimal cell population (middle). A specific molecular density surface provides optimal, homogenous cell populations (right), a possibility owed to the information gathered on the gradient

Nano-gradient technology offers a platform with an extensive range of biomolecule binding possibilities, providing a broad potential to gain knowledge of differentiation and cell-protein interactions. Moreover, the technology provides new opportunities to elucidate dose-dependent events, such as inducing migratory behavior. The nano-gradients are gradients of activator molecules bound to gold nanoparticles precisely distributed on a surface. They provide a unique chemically and physically defined substrate for controlled culture systems with a highly reproducible capacity (Andreasson et al., 2020a; Andreasson et al., 2020b; Lundgren et al., 2014; Evenbratt et al., 2020). One purpose of using gold nanoparticles is to present growth factors in a controlled manner to the cells. As the cells are immobilized on a surface, stimulations are comparable to *in vivo* conditions with matrix-bound cells, where local concentrations influence them (Fig. 1). These precise and stable molecular gradients enable dictating cell responses during differentiation because of the defined surface composition, density, and slope on a nano-level (Andreasson et al., 2020a; Andreasson et al., 2020b; Lundgren et al., 2014; Evenbratt et al., 2020).

The nano-gradients also allow the opportunity to combine factors, e.g., a growth factor with an ECM protein, further mimicking *in vivo* conditions, combining other materials and technologies, and forming a step in the differentiation protocol from where the cells can be removed and further cultured. The nano-gradient technology allows for screening an optimal growth factor density providing a robust differentiation protocol due to a precise and controlled stimulation, compared to, e.g., a solution-based gradient where growth factors are constantly moving (Minchiotti et al., 2006). Involving the gradient in differentiation protocols to generate chondrocyte progenitors could improve the ability to yield a defined cell population for differentiation before implantation into a damaged cartilage area (Andreasson et al., 2020a). However, further research is needed.

All stem cell research and therapeutic applications, such as tissue regeneration, require defined and stable protocols to precisely control the cells during differentiation, but also to maintain required cellular properties and simultaneously mimic *in vivo* conditions. Stem cell cultures for therapy require high cell quality and a homogeneous cell population; however, traditional 2D cultures provide limited expansion and differentiation capacity (Zhang et al., 2004). As mentioned, concentration gradients *in vivo* enable regulation of cell responses, which are necessary for the function and structure during tissue generation in embryonic development (Zecca et al., 1996; Peret & Murphy, 2008). Such gradients are essential inducers of many developmental and articular cartilage-generating processes.

## Conclusion

Current treatments of local cartilage lesions and OA focus on reducing pain and inflammation with insufficient long-term results. Today, no treatment is focused on disease-modifying mechanisms, and cell-based therapies struggle to generate high-quality cartilage. MSCs have become a commonly used cell source in developing approved and generally accepted stem cell therapy. Many companies have ongoing or completed clinical trials with promising results despite possible drawbacks, such as MSCs tending to form hypertrophic chondrocytes and bone instead of high-quality hyaline cartilage during chondrogenic differentiation. iPSC-derived chondrocytes have emerged as a potential alternative to MSCs, overcoming many of their drawbacks. However, issues, such as safety, have not been fully investigated to successfully commercialize iPSC-based treatments. To our knowledge, only one iPSC-based therapy for OA is in the clinical phase, currently undergoing a significant phase III trial. Biomolecular gradients are a potential aid to overcome problems with the differentiation of iPSCs. Gradients are essential in embryonic development. By utilizing gradients in the differentiation protocols, it is possible to provide a defined molecular stimulation to the cells and increase robustness compared to earlier protocols. A stable and more robust gradient would theoretically aid in generating a defined cell population for implantation into the damaged cartilage area. Further research, however, is required to accomplish this. Nonetheless, the research and development in this area are rapidly evolving in the quest to use stem cell-based therapies to treat cartilage damage.

## Data Availability

Not applicable.

## Abbreviations

OA: Osteoarthritis
iPSCs: Induced pluripotent stem cells
ECM: Extracellular matrix
ACI: Autologous chondrocyte implantation
MACI: Matrix-assisted chondrocyte implantation
3D: Three-dimensional
MSCs: Mesenchymal stem cells
ESCs: Embryonic stem cells
BMS: Bone marrow stimulation
PJAC: Particulated juvenile allograft cartilage
qPCR: Quantitative polymerase chain reaction
FACS: Fluorescent-activated cell sorting
RCT: Randomized controlled trials
